# Lysosomes and α-synuclein form a dangerous duet leading to neuronal cell death

**DOI:** 10.3389/fnana.2014.00083

**Published:** 2014-08-14

**Authors:** Mathieu Bourdenx, Erwan Bezard, Benjamin Dehay

**Affiliations:** ^1^Institut des Maladies Neurodégénératives, Université de Bordeaux, UMR 5293Bordeaux, France; ^2^CNRS, Institut des Maladies Neurodégénératives, UMR 5293Bordeaux, France

**Keywords:** α-synuclein, lysosome, Parkinson’s disease, cell death

## Abstract

Neurodegenerative diseases are (i) characterized by a selective neuronal vulnerability to degeneration in specific brain regions; and (ii) likely to be caused by disease-specific protein misfolding. Parkinson’s disease (PD) is characterized by the presence of intraneuronal proteinacious cytoplasmic inclusions, called Lewy Bodies (LB). α-Synuclein, an aggregation prone protein, has been identified as a major protein component of LB and the causative for autosomal dominant PD. Lysosomes are responsible for the clearance of long-lived proteins, such as α-synuclein, and for the removal of old or damaged organelles, such as mitochondria. Interestingly, PD-linked α-synuclein mutants and dopamine-modified wild-type α-synuclein block its own degradation, which result in insufficient clearance, leading to its aggregation and cell toxicity. Moreover, both lysosomes and lysosomal proteases have been found to be involved in the activation of certain cell death pathways. Interestingly, lysosomal alterations are observed in the brains of patients suffering from sporadic PD and also in toxic and genetic rodent models of PD-related neurodegeneration. All these events have unraveled a causal link between lysosomal impairment, α-synuclein accumulation, and neurotoxicity. In this review, we emphasize the pathophysiological mechanisms connecting α-synuclein and lysosomal dysfunction in neuronal cell death.

## Introduction

Neurodegenerative diseases are (i) characterized by a selective neuronal vulnerability to degeneration in specific brain regions; and (ii) likely caused by disease-specific protein misfolding. Parkinson’s disease (PD), the second most common neurodegenerative disorder after Alzheimer’s disease, is notably characterized by the loss of dopaminergic neurons in the substantia nigra pars compacta (SNpc). Loss of dopamine perturbates the cortico-basal ganglia-cortical control of voluntary movements. Current treatments have no proven protective or restorative effect and are only symptomatic. Moreover, their long-term use is associated with the onset of dramatic side effects i.e., fluctuated responses and L-Dopa induced dyskinesia. The important of understanding the mechanisms of neuronal death underlying neurodegenerative diseases is crucial for identifying targets for disease-modifying/curative strategies. In addition to dopaminergic neuronal cell loss, the main pathological hallmark of PD is the presence of intraneuronal proteinaceous cytoplasmic inclusions, named Lewy bodies (LB). α-Synuclein (α-syn), a major protein component of LB, has been identified as autosomal dominant cause of PD, which is found increased in expression in patients (Goedert et al., 2013; Lashuel et al., 2013). The presence of LB in PD suggests that defective protein handling contributes to the pathogenesis of the disease. Proteasomal and autophagic proteolysis are the two major pathways for degradation of cellular constituents in eukaryotic cells. Mounting evidence indicates that alterations in autophagy-lysosomal pathways (ALP) may be preferentially involved in PD. In this article, we review the close relationship between α-syn and the lysosome, two players involved in neuronal cell death in PD.

### The harmful α-synuclein

α-Syn has a central role in the pathogenesis of PD and other synucleinopathies, as dementia with Lewy bodies (DLB) and Multiple System Atrophy (MSA; Spillantini and Goedert, 2000). In 1997, the first link between PD and α-syn was described with the identification of point mutations -A53T- in the *SNCA* gene in autosomal-dominant forms of PD (Polymeropoulos et al., 1997; Athanassiadou et al., 1999; Spira et al., 2001; Ki et al., 2007; Choi et al., 2008; Puschmann et al., 2009). To date, the list of missense mutations continues to grow with A30P, E46K, H50Q, G51D, A53E (all classified as *PARK1* locus) (Krüger et al., 1998; Zarranz et al., 2004; Appel-Cresswell et al., 2013; Lesage et al., 2013; Proukakis et al., 2013; Pasanen et al., 2014). The subsequent identification of families with multiplication (duplication or triplication) of its allele (*PARK4* locus) strengthen the link between α-syn and PD (Singleton et al., 2003; Chartier-Harlin et al., 2004), suggesting that increased expression levels of the normal α-syn can be causal for PD and others synucleinopathies. Furthermore, genome-wide association studies (GWAS) have linked single nucleotide polymorphisms (SNPs) in the *SNCA* gene with increased susceptibility of developing PD (Simón-Sánchez et al., 2009; Edwards et al., 2010; International Parkinson Disease Genomics et al., 2011). α-Syn is a 14 kDa neuronal protein consisting of 140 amino acids mainly localized to presynaptic terminals. While the exact physiological function of α-syn remains to be fully understood, several studies have implicated its capacity to interact directly with cellular membranes, such as vesicles (Auluck et al., 2010) or mitochondria-associated membrane, which is an endoplasmic reticulum subdomain involved in lipid and calcium homeostasis (Guardia-Laguarta et al., 2014). Nevertheless, substantial evidence suggests that α-syn function is related to vesicle dynamics, neurotransmission and synaptic plasticity, the mechanisms of which have been reviewed elsewhere (Bellani et al., 2010). In its native state, the previous paradigm was that α-syn behaves as an unfolded monomer. However, a recent report now hints at a more complex picture as the predominant physiological species of α-syn is a helically folded tetramer (Bartels et al., 2011). α-Syn is, however, intrinsically defined as an aggregation-prone protein. In PD brains, α-syn antibodies strongly react in LB (Spillantini et al., 1997) and Lewy neurites (Takeda et al., 1998). Biochemical analyses have shown that α-syn is a major protein component of LB and may be part of the β-sheet enriched fibrillar structure of these inclusions (Crowther et al., 2000). α-Syn can undergo several post-translational modification such as truncation, nitration, oxidation, sumoylation, ubiquitinylation and phosphorylation (Giasson et al., 2000; Fujiwara et al., 2002; Tofaris et al., 2003; Anderson et al., 2006; Dorval and Fraser, 2006; Krumova et al., 2011). Interestingly, post-translationally modified α-syn has been found in LB and some post-translational modifications, such as oxidation or nitration, have been shown to impact its aggregation process in favor to oligomeric species (Fujiwara et al., 2002; Norris et al., 2003; Yamin et al., 2003). In the past few years, substantial progress has been made not only at elucidating how α-syn undergoes spontaneous self-aggregation, but also in its highly heterogenous aggregation process that turns its monomers into multiple oligomeric forms, then protofibrils, fibrils and aggregates. The identification of pathological species of α-syn involved in the perturbation of cellular function is an expanding area of research. Recent studies support the concept of soluble oligomers as the prominent toxic α-syn species in *in vitro* and *in vivo* settings, although the precise size and type of the toxic oligomeric species remains to be determined (Auluck et al., 2010; Winner et al., 2011; Cremades et al., 2012). Recent evidence piles up for prion-like propagation mechanisms in synucleinopathies, including PD. Indeed, α-syn might behave as a prion, responsible for initiating and spreading the pathological process in PD. Supporting this concept, α-syn can be transmitted to neighboring neurons and neuronal precursor cells (Puschmann et al., 2009; Hansen et al., 2011). *In vivo* studies have added a further piece to the puzzle with the observation that intracerebral inoculation of synthetic recombinant α-syn fibrils (Pffs) can mimic α-syn pathology in mice (Luk et al., 2012). More recently, through an innovative strategy based on the purification of aggregated α-syn from the SNpc of PD patients, intranigral or intrastriatal inoculations of PD-derived LB extracts resulted in progressive nigrostriatal neurodegeneration in both mice and monkeys (Recasens et al., 2014), which were found to originate at striatal dopaminergic terminals, Overall, these results demonstrated that human α-syn species contained in PD-derived LB are pathogenic and have the capacity to initiate a PD-like pathological process, not only in rodents but also in non-human primates (Recasens et al., 2014). Taken together, α-syn has multiple ways to cause cellular perturbations and lead to cell death. The presence of undegraded proteinaceous inclusions led the research community to wonder how is handled α-syn degradation? It is now understood that this involves both the ubiquitin-proteasome system (UPS) and the ALP. α-Syn is, however, predominantly degraded inside lysosomes, through chaperone-mediated autophagy (CMA) or endocytosis (Webb et al., 2003; Cuervo et al., 2004; Martinez-Vicente and Vila, 2013). The signals responsible for targeting α-syn (although it contains a KFERQ-like sequence, i.e., a motif recognized by heat shock cognate70 (hsc70) allowing direct lysosomal import) to a given degradation pathway are not yet fully understood, but may heavily depend on its folding state. Aggregated proteins will be preferentially routed for degradation to the lysosome through macroautophagy, whereas soluble forms would be both targeted to the proteasome or to the CMA. Overall, defective α-syn protein degradation can be recognized as an important pathogenic factor.

### Lysosome: white knight or two-face

Lysosomes are dynamic acidic organelles that contain hydrolytic enzyme capable of degrading intracellular components, which were discovered by Christian de Duve more than 50 years ago (De Duve et al., 1955; Luzio et al., 2007). Acidic pH (around 4.6) is maintained in the lumen by proton-pumping vacuolar ATPases. Around 200 proteins have been reported as lysosomal membrane proteins such as proton pumps, secretory, plasma membrane, signaling or transport proteins (Schröder et al., 2007). The most abundant proteins are the lysosomal-associated membrane protein (LAMP)-1 and LAMP-2 as well as the lysosomal integral membrane protein (LIMP)-2 and CD63 (Saftig et al., 2010). Interestingly, lysosomes have a high intravesicular Ca^2+^ concentration (around 500-600 μM). Defective lysosomal Ca^2+^ uptake has been associated with human diseases, such as Niemann-Pick type C (Lloyd-Evans and Platt, 2011). Several lysosomal storage disorders are caused by lysosomal membrane dysfunctions (Ruivo et al., 2009). These defects are mostly due to non-enzymatic transport defects, highlighting the importance of transport and channel proteins in lysosome physiology (Ruivo et al., 2009). As mitochondrial outer membrane permeabilization (MOMP) is a major checkpoint of apoptosis pathway, lysosomal membrane permeabilization (LMP) has also been shown to induce cell death (Boya and Kroemer, 2008). Following LMP, cell death can occur through several pathway including canonical MOMP/caspase pathway but also MOMP- and caspase-independent pathways (Boya and Kroemer, 2008). The two main effects of LMP are the release of lysosomal proteases, such as cathepsins B or D (CSTB/CTSD), and cytosolic acidification. CTSD or CTSB could then directly or indirectly promote cytochrome C release from mitochondria (Boya and Kroemer, 2008). Currently, the principal inducer of LMP remains to be reactive oxygen species (ROS), although Bcl-2-associated X protein (Bax) has also shown to initiate this process (Kågedal et al., 2005).

Several pathways converge to the lysosome: phagocytosis, endocytosis, and autophagy through three different means respectively named microautophagy, CMA and macroautophagy. Autophagy (which comes from the Greek: “self-eating”) is an evolutionary conserved mechanism that allows cells to degrade their own components and recycle important molecules (Wong and Cuervo, 2010; Cuervo, 2011; Boya et al., 2013). Briefly, CMA involves selective recognition by a chaperone and import through LAMP-2a, while microautophagy and macroautophagy involve direct sequestration of a portion of the cytosol (including proteins and organelles). While microautophagy requires the direct invagination of lysosomal membrane, macroautphagy involves the formation of a vesicle named autophagosome that will then fused with lysosomes to allow degradation of the sequestered material. In regards to protein aggregation, macroautophagy has been suggested to be the mammalian counterpart of the cytosolic-to-vacuole (Cvt)-pathway in yeast responsible for cargo-selective degradation (Yamamoto and Simonsen, 2011). Selective degradation of protein aggregates, named aggrephagy, has been characterized based on the observation of autophagosomes specifically containing aggregates (Filimonenko et al., 2010). Moreover, a phosphatidylinositol 3-phosphate-binding protein, Alfy, has been shown to specifically recognize and promote degradation of huntingtin aggregates (Filimonenko et al., 2010). For several decades, lysosomes have been only considered as terminal degradative compartments. However, recent studies suggest that lysosomes are involved in a vast number of cellular functions including lysosome-to-nucleus signaling, secretion, energy metabolism and cell death pathways (Rodriguez et al., 1997; Settembre et al., 2012, 2013).

Impairment of ALP, which is essential to maintain proper protein and organelle quantity and quality within cells, is increasingly regarded as a major pathogenic event in neurodegenerative diseases, including PD. The presence of LB in brains of PD patients made the first connection with ALP and led to the hypothesis that defective protein handling system might contribute to the pathogenesis of the disease. Several studies from independent groups reported ALP impairment associated with lysosomal depletion in brain tissue from idiopathic PD patients (Chu et al., 2009; Alvarez-Erviti et al., 2010; Dehay et al., 2010). More precisely, accumulation of undegraded microtubule-associated protein light chain 3 (LC3)-positive vesicles, decreased cytosolic hsc70, LAMP-1 and LAMP-2a have been reported (Chu et al., 2009; Alvarez-Erviti et al., 2010; Dehay et al., 2010). Genetic studies further strengthen the connection between PD and ALP dysfunction, which have indicated that lysosomal impairments may play a primary pathogenic role in the disease process. Interestingly, both CMA and proteasome can degrade the two proteins associated with autosomal dominant inheritance of PD, i.e., α-syn (*PARK1/PARK4* locus) and Leucine-rich repeat kinase 2 (LRRK2–*PARK8* locus) (Webb et al., 2003; Cuervo et al., 2004; Orenstein et al., 2013). However, PD-linked α-syn mutants (as well as post-translationally dopamine-modified wild-type α-syn) and mutant forms of LRRK2 block CMA activity, resulting in insufficient clearance and subsequent accumulation and aggregation of α-syn (Cuervo et al., 2004; Martinez-Vicente et al., 2008; Mak et al., 2010; Orenstein et al., 2013). Notably, two other genes encoding for lysosomal proteins have been linked to PD: the lysosomal type 5 P-type ATPase (ATP13A2—*PARK9* locus) (Ramirez et al., 2006) and the enzyme glucocerebrosidase (GBA; Aharon-Peretz et al., 2004; Di Fonzo et al., 2007; Sidransky et al., 2009). While the former has been characterized in rare families with prominent parkinsonism (Ramirez et al., 2006; Di Fonzo et al., 2007), the latter has been identified as risk factor in multicenter genetic analysis of patients (Sidransky et al., 2009). Recently, genetic analysis suggested that lysosomal dysfunction may play an important role in the etiology of DLB (Bras et al., 2014). Relevant to PD, these two proteins have been reported to be components of LB (Goker-Alpan et al., 2010; Dehay et al., 2012). Defects in one of these two proteins may result in insufficient clearance of α-syn through lysosomes, hence leading to the accumulation of this protein in both cytosol and lysosome lumen (Dehay et al., 2012). Such vicious pathogenic loop has been reported between GBA and α-syn (Mazzulli et al., 2011). One can thus imagine a similar scenario in which toxic species of α-syn “damage” lysosomes, hence leading to an impairment of α-syn clearance that subsequently favor α-syn-aggregation. Such aggregates then cause, in return, other damages, while concomitantly accumulating in lysosomes/autolysosomes to form LB (Dehay et al., 2012).

In addition to the aforementioned genes, hereditary parkinsonism has been identified in families carrying mutations for ALP-related pathways. For instance, mutations in parkin (*PARK2* locus), in the phosphatase and tensin homolog (PTEN)-induced putative kinase 1 (PINK1) (*PARK6* locus) or in DJ-1 (*PARK7* locus), which are all involved in mitophagy, lead to autosomal recessive forms of PD (Corti et al., 2011). While PINK1 and parkin belong to the same pathway, DJ-1 has been shown to be involved in an independent parallel pathway, which can rescue a loss of function of PINK1 (Hao et al., 2010; Thomas et al., 2011). A defective degradation of dysfunctional mitochondria leads to maintaining those in the neuron and hence promotes the mitochondrial dysfunctions that have been characterized in PD patients (i.e., decrease in complex I activity and accumulation of large-scale mitochondrial DNA mutations) (Schapira et al., 1989; Bender et al., 2006). Mutations in the PD-associated gene *UCH-L1* (*PARK5*) abnormally interact with LAMP-2A, also causing an increase amount of α-syn (Kabuta et al., 2008). From a genetic point of view, all genes that have been positively associated with PD (Corti et al., 2011) are also connected to ALP, which shed light on the lysosome as an important player in PD-induced cell death.

### Neuronal cell death: the third partner

Lysosomal function impairment and α-syn aggregation can both induce cell death either on their own or through a dramatic additive effect. Of importance, α-syn seems to induce cell toxicity through its different pathological α-syn species, which include post-translationally modified, mutant, oligomeric and aggregated forms. These can (i) disrupt its typical function in neurotransmission release (Abeliovich et al., 2000; Jenco et al., 1998); (ii) impair mitochondrial dynamics, structure and function (Martin et al., 2006; Nakamura et al., 2011; Stefanovic et al., 2014); and (iii) disrupt ER-Golgi vesicle trafficking (Cooper et al., 2006; Gitler et al., 2008) and mitochondria-associated ER membrane (Mercado et al., 2013; Guardia-Laguarta et al., 2014), which results in ER stress. Further supporting the α-syn species toxicity, CMA inhibition by either PD-linked α-syn mutants or dopamine-modified wild-type α-syn results in an accumulation of α-syn, but also of undegraded CMA-substrates, involved for instance in the regulation of neuronal survival through the degradation of the neuronal survival factor myocyte enhancer factor 2D (MEF2D; Yang et al., 2009).

Regarding the lysosome, LMP is one mechanism for the induction of certain cell death pathways. As mentioned above, disruption of lysosomal membrane provokes cell death through release of CTSs and other hydrolases from the lysosomal lumen to the cytosol. These lysosomal proteases can remain active at cytosolic pH and induce cellular damages by degradation of vital proteins or activation of caspases. In relation to PD pathophysiology, mechanistic studies using the 1-methyl-4-phenyl-1,2,3,6-tetrahydropyridine (MPTP) mouse model of PD have reported a lysosomal dysfunction, characterized by lysosomal depletion and autophagosome accumulation. Such lysosomal deficiency was secondary to abnormal LMP induced by Complex I inhibition-mediated ROS production (Dehay et al., 2010; Vila et al., 2011). Recent studies reported that the pro-apoptotic Bax protein, which mediates MOMP, is activated in PD patients (Bové et al., 2014). In experimental PD mouse model, Bax translocates to the lysosome and mediates LMP before MOMP (Bové et al., 2014). Interestingly, pharmacological inhibition of Bax-mediated LMP and MOMP results in an overall attenuation of MPTP-mediated cell death, even if the treatment is administered once pathogenic neuronal changes are already in motion (Bové et al., 2014), suggesting that the phenomenon at work is reversible.

One of the meeting points between α-syn and lysosome involves ROS. Recent reports suggest that α-syn oligomers can induce both MOMP and in particular LMP (Freeman et al., 2013; Stefanovic et al., 2014). α-Syn aggregation underlies a bidirectional relationship with ROS production. Specific α-syn oligomers increase ROS production, whereas oxidized α-syn inhibits fibril formation in favor to toxic species (Norris et al., 2003; Cremades et al., 2012). Hence, α-syn-mediated ROS production can lead to LMP, as previously characterized in PD, and subsequently to cell death. All these studies suggest that oxidative stress impact both lysosomes and α-syn aggregation. In the past few years, another piece has been added to the puzzle, suggesting that α-syn might potentially spread in a prion-like manner, from cell to cell and region to region. Although mechanisms of α-syn release are not yet elucidated, α-syn may be released by exocytosis in a calcium-dependent manner (Lee et al., 2005; Emmanouilidou et al., 2010), a phenomenon exacerbated after lysosomal inhibition (Alvarez-Erviti et al., 2011), subsequently enhancing disease progression and the lysosomal contribution to the pathology. Non-genetic factors, however, cannot be excluded as important risks to PD. This includes ageing for instance which remains the most compelling risk factor for PD. Ageing is also associated with mitochondrial and lysosomal impairments as well as ROS production (Dufour and Larsson, 2004; Mattson and Magnus, 2006), linking the several key events that occur in neuronal cell death in PD.

Of interest, pharmacological or genetic enhancement of autophagy has been shown to be beneficial in PD models. For example, in the MPTP-treated mouse model, pharmacological activation of ALP with the mammalian target of rapamycin (mTOR) inhibitor, rapamycin, attenuates neurodegeneration and lysosomal dysfunction (Dehay et al., 2010; Malagelada et al., 2010). Consistent with this approach, viral-mediated overexpression of ALP components, such as transcription factor EB (TFEB), LAMP2a or Beclin-1, provided neuroprotection in viral-mediated α-syn-overexpressing rodent models of PD (Spencer et al., 2009; Decressac et al., 2013; Xilouri et al., 2013). With regards to the development of therapeutic approaches, we must keep in mind that a balance needs to be maintained between boosting and inhibiting processes of autophagy. Indeed, autophagy has been shown to have both survival promoting and death promoting roles (Eskelinen, 2005). Hence, enhancement of lysosomal biogenesis or specific activation of late steps of the autophagy machinery might provide more successful approach compared to a broad activation of the whole autophagy machinery, potentially leading to a deleterious effect and eventually cell death. Increasing the ability of neurons under attack to degrade protein aggregates remains a promising strategy for PD.

## Concluding remarks

**Figure 1:**
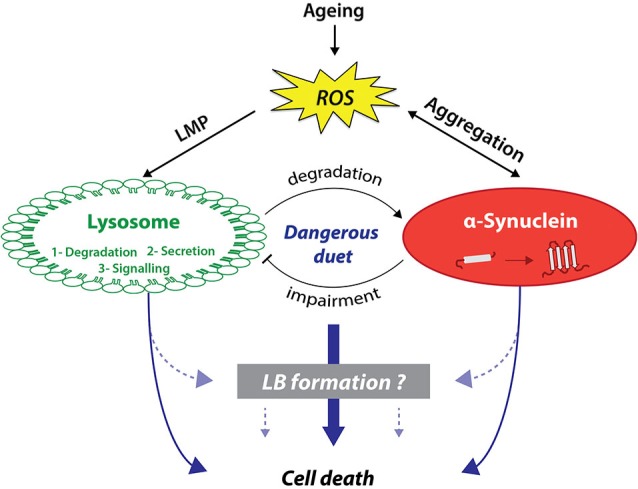
**Lysosomes and α-synuclein are involved in a vicious pathogenic loop eventually leading to cell death and LB formation**. On the one hand, lysosomes have been shown to be involved in cell death activation through canonical or non-canonical pathways. On the other hand, α-synuclein (α-syn) can also trigger cell death through several distinct pathways including membranes permeabilization. Lysosomes and α-syn display a bidirectional relationship. While lysosomes predominantly degrade α-syn, α-syn aggregation can lead to lysosomal dysfunction in return. α-Syn-mediated lysosome impairment can lead to alterations of one of lysosomal main function: protein degradation, lysosome-to-nucleus signaling, and secretion. This pathogenic loop can be worsened with age and in particular ROS production, which can induce both LMP and α-syn aggregation. Interestingly, α-syn aggregation, especially specific oligomeric species, can increase ROS production. We previously suggested that this loop might be the template for the formation of LB, which remains currently unknown.

Seventeen years after its association with PD, α-syn is now considered as a central player in PD pathogenesis, linking genetic and idiopathic forms of parkinsonism. Two key elements strongly associate α-syn aggregation and lysosomal dysfunction: (i) aggregated or post-translationally modified forms of α-syn can directly or indirectly inhibit lysosomal function; and (ii) the occurrence of a lysosomal depletion in brains from PD patients as well as in several experimental models of PD. Consistent with these evidences, LB formation might be the result of the combination of both α-syn aggregation and lysosomal failure, as key components of autophagy and α-syn have been localized in LB. Altogether, this suggests that α-syn aggregation and lysosomal impairment, enhanced with ageing, could play a deleterious duet leading to dopaminergic cell death (Figure 1).

## Conflict of interest statement

The authors declare that the research was conducted in the absence of any commercial or financial relationships that could be construed as a potential conflict of interest.

## References

[B97] Abeliovich A. Schmitz Y. Farinas I. Choi-Lundberg D. Ho W. H. Castillo P. E. (2000). Mice lacking alpha-synuclein display functional deficits in the nigrostriatal dopamine system. Neuron 25, 239–252 10.1016/s0896-6273(00)80886-7 10707987

[B1] Aharon-Peretz J. Rosenbaum H. Gershoni-Baruch R. (2004). Mutations in the glucocerebrosidase gene and Parkinson’s disease in Ashkenazi Jews. N. Engl. J. Med. 351, 1972–1977 10.1056/nejmoa033277 15525722

[B2] Alvarez-Erviti L. Rodriguez-Oroz M. C. Cooper J. M. Caballero C. Ferrer I. Obeso J. A. (2010). Chaperone-mediated autophagy markers in Parkinson disease brains. Arch. Neurol. 67, 1464–1472 10.1001/archneurol.2010.198 20697033

[B101] Alvarez-Erviti L. Seow Y. Schapira A. H. Gardiner C. Sargent I. L. Wood M. J. (2011). Lysosomal dysfunction increases exosome-mediated alpha-synuclein release and transmission. Neurobiol. Dis. 42, 360–367 10.1016/j.nbd.2011.01.029 21303699 PMC3107939

[B3] Anderson J. P. Walker D. E. Goldstein J. M. De Laat R. Banducci K. Caccavello R. J. (2006). Phosphorylation of Ser-129 is the dominant pathological modification of alpha-synuclein in familial and sporadic Lewy body disease. J. Biol. Chem. 281, 29739–29752 10.1074/jbc.m600933200 16847063

[B4] Appel-Cresswell S. Vilarino-Guell C. Encarnacion M. Sherman H. Yu I. Shah B. (2013). Alpha-synuclein p.H50Q, a novel pathogenic mutation for Parkinson’s disease. Mov. Disord. 28, 811–813 10.1002/mds.25421 23457019

[B5] Athanassiadou A. Voutsinas G. Psiouri L. Leroy E. Polymeropoulos M. H. Ilias A. (1999). Genetic analysis of families with Parkinson disease that carry the Ala53Thr mutation in the gene encoding alpha-synuclein. Am. J. Hum. Genet. 65, 555–558 10.1086/302486 10417297 PMC1377953

[B6] Auluck P. K. Caraveo G. Lindquist S. (2010). alpha-Synuclein: membrane interactions and toxicity in Parkinson’s disease. Annu. Rev. Cell Dev. Biol. 26, 211–233 10.1146/annurev.cellbio.042308.113313 20500090

[B7] Bartels T. Choi J. G. Selkoe D. J. (2011). alpha-Synuclein occurs physiologically as a helically folded tetramer that resists aggregation. Nature 477, 107–110 10.1038/nature10324 21841800 PMC3166366

[B8] Bellani S. Sousa V. L. Ronzitti G. Valtorta F. Meldolesi J. Chieregatti E. (2010). The regulation of synaptic function by alpha-synuclein. Commun. Integr. Biol. 3, 106–109 10.4161/cib.3.2.10964 20585500 PMC2889964

[B9] Bender A. Krishnan K. J. Morris C. M. Taylor G. A. Reeve A. K. Perry R. H. (2006). High levels of mitochondrial DNA deletions in substantia nigra neurons in aging and Parkinson disease. Nat. Genet. 38, 515–517 10.1038/ng1769 16604074

[B10] Bové J. Martinez-Vicente M. Dehay B. Perier C. Recasens A. Bombrun A. (2014). BAX channel activity mediates lysosomal disruption linked to Parkinson disease. Autophagy 10, 889–900 10.4161/auto.28286 24686337 PMC5119069

[B11] Boya P. Kroemer G. (2008). Lysosomal membrane permeabilization in cell death. Oncogene 27, 6434–6451 10.1038/onc.2008.310 18955971

[B12] Boya P. Reggiori F. Codogno P. (2013). Emerging regulation and functions of autophagy. Nat. Cell Biol. 15, 713–720 10.1038/ncb2788 23817233 PMC7097732

[B13] Bras J. Guerreiro R. Darwent L. Parkkinen L. Ansorge O. Escott-Price V. (2014). Genetic analysis implicates APOE, SNCA and suggests lysosomal dysfunction in the etiology of dementia with Lewy bodies. Hum. Mol. Genet. [Epub ahead of print]. 10.1093/hmg/ddu334 24973356 PMC4222357

[B14] Chartier-Harlin M. C. Kachergus J. Roumier C. Mouroux V. Douay X. Lincoln S. (2004). Alpha-synuclein locus duplication as a cause of familial Parkinson’s disease. Lancet 364, 1167–1169 10.1016/s0140-6736(04)17103-1 15451224

[B15] Choi J. M. Woo M. S. Ma H. I. Kang S. Y. Sung Y. H. Yong S. W. (2008). Analysis of PARK genes in a Korean cohort of early-onset Parkinson disease. Neurogenetics 9, 263–269 10.1007/s10048-008-0138-0 18704525

[B16] Chu Y. Dodiya H. Aebischer P. Olanow C. W. Kordower J. H. (2009). Alterations in lysosomal and proteasomal markers in Parkinson’s disease: relationship to alpha-synuclein inclusions. Neurobiol. Dis. 35, 385–398 10.1016/j.nbd.2009.05.023 19505575

[B17] Cooper A. A. Gitler A. D. Cashikar A. Haynes C. M. Hill K. J. Bhullar B. (2006). Alpha-synuclein blocks ER-Golgi traffic and Rab1 rescues neuron loss in Parkinson’s models. Science 313, 324–328 10.1126/science.1129462 16794039 PMC1983366

[B18] Corti O. Lesage S. Brice A. (2011). What genetics tells us about the causes and mechanisms of Parkinson’s disease. Physiol. Rev. 91, 1161–1218 10.1152/physrev.00022.2010 22013209

[B19] Cremades N. Cohen S. I. Deas E. Abramov A. Y. Chen A. Y. Orte A. (2012). Direct observation of the interconversion of normal and toxic forms of alpha-synuclein. Cell 149, 1048–1059 10.1016/j.cell.2012.03.037 22632969 PMC3383996

[B20] Crowther R. A. Daniel S. E. Goedert M. (2000). Characterisation of isolated alpha-synuclein filaments from substantia nigra of Parkinson’s disease brain. Neurosci. Lett. 292, 128–130 10.1016/s0304-3940(00)01440-3 10998565

[B21] Cuervo A. M. (2011). Cell biology. Autophagy’s top chef. Science 332, 1392–1393 10.1126/science.1208607 21680833

[B22] Cuervo A. M. Stefanis L. Fredenburg R. Lansbury P. T. Sulzer D. (2004). Impaired degradation of mutant alpha-synuclein by chaperone-mediated autophagy. Science 305, 1292–1295 10.1126/science.1101738 15333840

[B24] Decressac M. Mattsson B. Weikop P. Lundblad M. Jakobsson J. Bjorklund A. (2013). TFEB-mediated autophagy rescues midbrain dopamine neurons from alpha-synuclein toxicity. Proc. Natl. Acad. Sci. U S A 110, E1817–E1826 10.1073/pnas.1305623110 23610405 PMC3651458

[B23] De Duve C. Pressman B. C. Gianetto R. Wattiaux R. Appelmans F. (1955). Tissue fractionation studies. 6. Intracellular distribution patterns of enzymes in rat-liver tissue. Biochem. J. 60, 604–617 13249955 10.1042/bj0600604 PMC1216159

[B25] Dehay B. Bové J. Rodriguez-Muela N. Perier C. Recasens A. Boya P. (2010). Pathogenic lysosomal depletion in Parkinson’s disease. J. Neurosci. 30, 12535–12544 10.1523/jneurosci.1920-10.2010 20844148 PMC6633458

[B26] Dehay B. Ramirez A. Martinez-Vicente M. Perier C. Canron M. H. Doudnikoff E. (2012). Loss of P-type ATPase ATP13A2/PARK9 function induces general lysosomal deficiency and leads to Parkinson disease neurodegeneration. Proc. Natl. Acad. Sci. U S A 109, 9611–9616 10.1073/pnas.1112368109 22647602 PMC3386132

[B27] Di Fonzo A. Chien H. F. Socal M. Giraudo S. Tassorelli C. Iliceto G. (2007). ATP13A2 missense mutations in juvenile parkinsonism and young onset Parkinson disease. Neurology 68, 1557–1562 10.1212/01.wnl.0000260963.08711.08 17485642

[B28] Dorval V. Fraser P. E. (2006). Small ubiquitin-like modifier (SUMO) modification of natively unfolded proteins tau and alpha-synuclein. J. Biol. Chem. 281, 9919–9924 10.1074/jbc.m510127200 16464864

[B29] Dufour E. Larsson N. G. (2004). Understanding aging: revealing order out of chaos. Biochim. Biophys. Acta 1658, 122–132 10.1016/j.bbabio.2004.04.020 15282183

[B30] Edwards T. L. Scott W. K. Almonte C. Burt A. Powell E. H. Beecham G. W. (2010). Genome-wide association study confirms SNPs in SNCA and the MAPT region as common risk factors for Parkinson disease. Ann. Hum. Genet. 74, 97–109 10.1111/j.1469-1809.2009.00560.x 20070850 PMC2853717

[B100] Emmanouilidou E. Melachroinou K. Roumeliotis T. Garbis S. D. Ntzouni M. Margaritis L. H. (2010). Cell-produced alpha-synuclein is secreted in a calcium-dependent manner by exosomes and impacts neuronal survival. J. Neurosci. 30, 6838–6851 10.1523/JNEUROSCI.5699-09.2010 20484626 PMC3842464

[B31] Eskelinen E. L. (2005). Doctor Jekyll and Mister Hyde: autophagy can promote both cell survival and cell death. Cell Death Differ. 12(Suppl. 2), 1468–1472 10.1038/sj.cdd.4401721 16247492

[B32] Filimonenko M. Isakson P. Finley K. D. Anderson M. Jeong H. Melia T. J. (2010). The selective macroautophagic degradation of aggregated proteins requires the PI3P-binding protein Alfy. Mol. Cell 38, 265–279 10.1016/j.molcel.2010.04.007 20417604 PMC2867245

[B33] Freeman D. Cedillos R. Choyke S. Lukic Z. Mcguire K. Marvin S. (2013). Alpha-synuclein induces lysosomal rupture and cathepsin dependent reactive oxygen species following endocytosis. PLoS One 8:e62143 10.1371/journal.pone.0062143 23634225 PMC3636263

[B34] Fujiwara H. Hasegawa M. Dohmae N. Kawashima A. Masliah E. Goldberg M. S. (2002). alpha-Synuclein is phosphorylated in synucleinopathy lesions. Nat. Cell Biol. 4, 160–164 10.1038/ncb748 11813001

[B35] Giasson B. I. Duda J. E. Murray I. V. Chen Q. Souza J. M. Hurtig H. I. (2000). Oxidative damage linked to neurodegeneration by selective alpha-synuclein nitration in synucleinopathy lesions. Science 290, 985–989 10.1126/science.290.5493.985 11062131

[B36] Gitler A. D. Bevis B. J. Shorter J. Strathearn K. E. Hamamichi S. Su L. J. (2008). The Parkinson’s disease protein alpha-synuclein disrupts cellular Rab homeostasis. Proc. Natl. Acad. Sci. U S A 105, 145–150 10.1073/pnas.0710685105 18162536 PMC2224176

[B37] Goedert M. Spillantini M. G. Del Tredici K. Braak H. (2013). 100 years of Lewy pathology. Nat. Rev. Neurol. 9, 13–24 10.1038/nrneurol.2012.242 23183883

[B38] Goker-Alpan O. Stubblefield B. K. Giasson B. I. Sidransky E. (2010). Glucocerebrosidase is present in alpha-synuclein inclusions in Lewy body disorders. Acta Neuropathol. 120, 641–649 10.1007/s00401-010-0741-7 20838799 PMC3352317

[B39] Guardia-Laguarta C. Area-Gomez E. Rüb C. Liu Y. Magrane J. Becker D. (2014). alpha-Synuclein is localized to mitochondria-associated ER membranes. J. Neurosci. 34, 249–259 10.1523/jneurosci.2507-13.2014 24381286 PMC3866487

[B40] Hansen C. Angot E. Bergstrom A. L. Steiner J. A. Pieri L. Paul G. (2011). alpha-Synuclein propagates from mouse brain to grafted dopaminergic neurons and seeds aggregation in cultured human cells. J. Clin. Invest. 121, 715–725 10.1172/JCI43366 21245577 PMC3026723

[B41] Hao L. Y. Giasson B. I. Bonini N. M. (2010). DJ-1 is critical for mitochondrial function and rescues PINK1 loss of function. Proc. Natl. Acad. Sci. U S A 107, 9747–9752 10.1073/pnas.0911175107 20457924 PMC2906840

[B42] International Parkinson Disease Genomics C. Nalls M. A. Plagnol V. Hernandez D. G. Sharma M. Sheerin U. M. (2011). Imputation of sequence variants for identification of genetic risks for Parkinson’s disease: a meta-analysis of genome-wide association studies. Lancet 377, 641–649 10.1016/S0140-6736(10)62345-8 21292315 PMC3696507

[B98] Jenco J. M. Rawlingson A. Daniels B. Morris A. J. (1998). Regulation of phospholipase D2: selective inhibition of mammalian phospholipase D isoenzymes by alpha- and beta-synucleins. Biochemistry 37, 4901–4909 10.1021/bi972776r 9538008

[B43] Kabuta T. Setsuie R. Mitsui T. Kinugawa A. Sakurai M. Aoki S. (2008). Aberrant molecular properties shared by familial Parkinson’s disease-associated mutant UCH-L1 and carbonyl-modified UCH-L1. Hum. Mol. Genet. 17, 1482–1496 10.1093/hmg/ddn037 18250096

[B44] Kågedal K. Johansson A. C. Johansson U. Heimlich G. Roberg K. Wang N. S. (2005). Lysosomal membrane permeabilization during apoptosis–involvement of Bax? Int. J. Exp. Pathol. 86, 309–321 10.1111/j.0959-9673.2005.00442.x 16191103 PMC2517437

[B45] Ki C. S. Stavrou E. F. Davanos N. Lee W. Y. Chung E. J. Kim J. Y. (2007). The Ala53Thr mutation in the alpha-synuclein gene in a Korean family with Parkinson disease. Clin. Genet. 71, 471–473 10.1111/j.1399-0004.2007.00781.x 17489854

[B46] Krüger R. Kuhn W. Müller T. Woitalla D. Graeber M. Kosel S. (1998). Ala30Pro mutation in the gene encoding alpha-synuclein in Parkinson’s disease. Nat. Genet. 18, 106–108 9462735 10.1038/ng0298-106

[B47] Krumova P. Meulmeester E. Garrido M. Tirard M. Hsiao H. H. Bossis G. (2011). Sumoylation inhibits alpha-synuclein aggregation and toxicity. J. Cell Biol. 194, 49–60 10.1083/jcb.201010117 21746851 PMC3135405

[B48] Lashuel H. A. Overk C. R. Oueslati A. Masliah E. (2013). The many faces of alpha-synuclein: from structure and toxicity to therapeutic target. Nat. Rev. Neurosci. 14, 38–48 10.1038/nrn3406 23254192 PMC4295774

[B99] Lee H. J. Patel S. Lee S. J. (2005). Intravesicular localization and exocytosis of alpha-synuclein and its aggregates. J. Neurosci. 25, 6016–6024 10.1523/jneurosci.0692-05.2005 15976091 PMC6724798

[B49] Lesage S. Anheim M. Letournel F. Bousset L. Honore A. Rozas N. (2013). G51D alpha-synuclein mutation causes a novel parkinsonian-pyramidal syndrome. Ann. Neurol. 73, 459–471 10.1002/ana.23894 23526723

[B50] Lloyd-Evans E. Platt F. M. (2011). Lysosomal Ca(2+) homeostasis: role in pathogenesis of lysosomal storage diseases. Cell Calcium 50, 200–205 10.1016/j.ceca.2011.03.010 21724254

[B51] Luk K. C. Kehm V. Carroll J. Zhang B. O’brien P. Trojanowski J. Q. (2012). Pathological alpha-synuclein transmission initiates Parkinson-like neurodegeneration in nontransgenic mice. Science 338, 949–953 10.1126/science.1227157 23161999 PMC3552321

[B52] Luzio J. P. Pryor P. R. Bright N. A. (2007). Lysosomes: fusion and function. Nat. Rev. Mol. Cell Biol. 8, 622–632 10.1038/nrm2217 17637737

[B53] Mak S. K. Mccormack A. L. Manning-Bog A. B. Cuervo A. M. Di Monte D. A. (2010). Lysosomal degradation of alpha-synuclein in vivo. J. Biol. Chem. 285, 13621–13629 10.1074/jbc.m109.074617 20200163 PMC2859524

[B54] Malagelada C. Jin Z. H. Jackson-Lewis V. Przedborski S. Greene L. A. (2010). Rapamycin protects against neuron death in in vitro and in vivo models of Parkinson’s disease. J. Neurosci. 30, 1166–1175 10.1523/jneurosci.3944-09.2010 20089925 PMC2880868

[B55] Martin L. J. Pan Y. Price A. C. Sterling W. Copeland N. G. Jenkins N. A. (2006). Parkinson’s disease alpha-synuclein transgenic mice develop neuronal mitochondrial degeneration and cell death. J. Neurosci. 26, 41–50 10.1523/jneurosci.4308-05.2006 16399671 PMC6381830

[B56] Martinez-Vicente M. Talloczy Z. Kaushik S. Massey A. C. Mazzulli J. Mosharov E. V. (2008). Dopamine-modified alpha-synuclein blocks chaperone-mediated autophagy. J. Clin. Invest. 118, 777–788 10.1172/JCI32806 18172548 PMC2157565

[B57] Martinez-Vicente M. Vila M. (2013). Alpha-synuclein and protein degradation pathways in Parkinson’s disease: a pathological feed-back loop. Exp. Neurol. 247, 308–313 10.1016/j.expneurol.2013.03.005 23499831

[B58] Mattson M. P. Magnus T. (2006). Ageing and neuronal vulnerability. Nat. Rev. Neurosci. 7, 278–294 10.1038/nrn1886 16552414 PMC3710114

[B59] Mazzulli J. R. Xu Y. H. Sun Y. Knight A. L. Mclean P. J. Caldwell G. A. (2011). Gaucher disease glucocerebrosidase and alpha-synuclein form a bidirectional pathogenic loop in synucleinopathies. Cell 146, 37–52 10.1016/j.cell.2011.06.001 21700325 PMC3132082

[B60] Mercado G. Valdés P. Hetz C. (2013). An ERcentric view of Parkinson’s disease. Trends Mol. Med. 19, 165–175 10.1016/j.molmed.2012.12.005 23352769

[B61] Nakamura K. Nemani V. M. Azarbal F. Skibinski G. Levy J. M. Egami K. (2011). Direct membrane association drives mitochondrial fission by the Parkinson disease-associated protein alpha-synuclein. J. Biol. Chem. 286, 20710–20726 10.1074/jbc.p110.213538 21489994 PMC3121472

[B62] Norris E. H. Giasson B. I. Ischiropoulos H. Lee V. M. (2003). Effects of oxidative and nitrative challenges on alpha-synuclein fibrillogenesis involve distinct mechanisms of protein modifications. J. Biol. Chem. 278, 27230–27240 10.1074/jbc.m212436200 12857790

[B63] Orenstein S. J. Kuo S. H. Tasset I. Arias E. Koga H. Fernandez-Carasa I. (2013). Interplay of LRRK2 with chaperone-mediated autophagy. Nat. Neurosci. 16, 394–406 10.1038/nn.3350 23455607 PMC3609872

[B64] Pasanen P. Myllykangas L. Siitonen M. Raunio A. Kaakkola S. Lyytinen J. (2014). A novel alpha-synuclein mutation A53E associated with atypical multiple system atrophy and Parkinson’s disease-type pathology. Neurobiol. Aging 35, 2180.e1–2180.e5 10.1016/j.neurobiolaging.2014.03.024 24746362

[B65] Polymeropoulos M. H. Lavedan C. Leroy E. Ide S. E. Dehejia A. Dutra A. (1997). Mutation in the alpha-synuclein gene identified in families with Parkinson’s disease. Science 276, 2045–2047 10.1126/science.276.5321.2045 9197268

[B66] Proukakis C. Dudzik C. G. Brier T. Mackay D. S. Cooper J. M. Millhauser G. L. (2013). A novel alpha-synuclein missense mutation in Parkinson disease. Neurology 80, 1062–1064 10.1212/wnl.0b013e31828727ba 23427326 PMC3653201

[B67] Puschmann A. Ross O. A. Vilarino-Guell C. Lincoln S. J. Kachergus J. M. Cobb S. A. (2009). A Swedish family with de novo alpha-synuclein A53T mutation: evidence for early cortical dysfunction. Parkinsonism Relat. Disord. 15, 627–632 10.1016/j.parkreldis.2009.06.007 19632874 PMC2783246

[B68] Ramirez A. Heimbach A. Gründemann J. Stiller B. Hampshire D. Cid L. P. (2006). Hereditary parkinsonism with dementia is caused by mutations in ATP13A2, encoding a lysosomal type 5 P-type ATPase. Nat. Genet. 38, 1184–1191 10.1038/ng1884 16964263

[B69] Recasens A. Dehay B. Bové J. Carballo-Carbajal I. Dovero S. Perez-Villalba A. (2014). Lewy body extracts from Parkinson disease brains trigger alpha-synuclein pathology and neurodegeneration in mice and monkeys. Ann. Neurol. 75, 351–362 10.1002/ana.24066 24243558

[B70] Rodriguez A. Webster P. Ortego J. Andrews N. W. (1997). Lysosomes behave as Ca2+-regulated exocytic vesicles in fibroblasts and epithelial cells. J. Cell Biol. 137, 93–104 10.1083/jcb.137.1.93 9105039 PMC2139854

[B71] Ruivo R. Anne C. Sagné C. Gasnier B. (2009). Molecular and cellular basis of lysosomal transmembrane protein dysfunction. Biochim. Biophys. Acta 1793, 636–649 10.1016/j.bbamcr.2008.12.008 19146888

[B72] Saftig P. Schröder B. Blanz J. (2010). Lysosomal membrane proteins: life between acid and neutral conditions. Biochem. Soc. Trans. 38, 1420–1423 10.1042/bst0381420 21118100

[B73] Schapira A. H. Cooper J. M. Dexter D. Jenner P. Clark J. B. Marsden C. D. (1989). Mitochondrial complex I deficiency in Parkinson’s disease. Lancet 1, 1269 2566813 10.1016/s0140-6736(89)92366-0

[B74] Schröder B. Wrocklage C. Pan C. Jäger R. Kösters B. Schäfer H. (2007). Integral and associated lysosomal membrane proteins. Traffic 8, 1676–1686 10.1111/j.1600-0854.2007.00643.x 17897319

[B75] Settembre C. Fraldi A. Medina D. L. Ballabio A. (2013). Signals from the lysosome: a control centre for cellular clearance and energy metabolism. Nat. Rev. Mol. Cell Biol. 14, 283–296 10.1038/nrm3565 23609508 PMC4387238

[B76] Settembre C. Zoncu R. Medina D. L. Vetrini F. Erdin S. Erdin S. (2012). A lysosome-to-nucleus signalling mechanism senses and regulates the lysosome via mTOR and TFEB. EMBO J. 31, 1095–1108 10.1038/emboj.2012.32 22343943 PMC3298007

[B77] Sidransky E. Nalls M. A. Aasly J. O. Aharon-Peretz J. Annesi G. Barbosa E. R. (2009). Multicenter analysis of glucocerebrosidase mutations in Parkinson’s disease. N. Engl. J. Med. 361, 1651–1661 10.1056/NEJMoa0901281 19846850 PMC2856322

[B78] Simón-Sánchez J. Schulte C. Bras J. M. Sharma M. Gibbs J. R. Berg D. (2009). Genome-wide association study reveals genetic risk underlying Parkinson’s disease. Nat. Genet. 41, 1308–1312 10.1038/ng.487 19915575 PMC2787725

[B79] Singleton A. B. Farrer M. Johnson J. Singleton A. Hague S. Kachergus J. (2003). alpha-Synuclein locus triplication causes Parkinson’s disease. Science 302, 841 10.1126/science.1090278 14593171

[B80] Spencer B. Potkar R. Trejo M. Rockenstein E. Patrick C. Gindi R. (2009). Beclin 1 gene transfer activates autophagy and ameliorates the neurodegenerative pathology in alpha-synuclein models of Parkinson’s and Lewy body diseases. J. Neurosci. 29, 13578–13588 10.1523/jneurosci.4390-09.2009 19864570 PMC2812014

[B81] Spillantini M. G. Goedert M. (2000). The alpha-synucleinopathies: Parkinson’s disease, dementia with Lewy bodies and multiple system atrophy. Ann. N Y Acad. Sci. 920, 16–27 10.1111/j.1749-6632.2000.tb06900.x 11193145

[B82] Spillantini M. G. Schmidt M. L. Lee V. M. Trojanowski J. Q. Jakes R. Goedert M. (1997). Alpha-synuclein in Lewy bodies. Nature 388, 839–840 10.1038/42166 9278044

[B83] Spira P. J. Sharpe D. M. Halliday G. Cavanagh J. Nicholson G. A. (2001). Clinical and pathological features of a Parkinsonian syndrome in a family with an Ala53Thr alpha-synuclein mutation. Ann. Neurol. 49, 313–319 10.1002/ana.67.abs 11261505

[B84] Stefanovic A. N. Stöckl M. T. Claessens M. M. Subramaniam V. (2014). alpha-Synuclein oligomers distinctively permeabilize complex model membranes. FEBS J. 281, 2838–2850 10.1111/febs.12824 24767583

[B85] Takeda A. Mallory M. Sundsmo M. Honer W. Hansen L. Masliah E. (1998). Abnormal accumulation of NACP/alpha-synuclein in neurodegenerative disorders. Am. J. Pathol. 152, 367–372 9466562 PMC1857971

[B86] Thomas K. J. Mccoy M. K. Blackinton J. Beilina A. Van Der Brug M. Sandebring A. (2011). DJ-1 acts in parallel to the PINK1/parkin pathway to control mitochondrial function and autophagy. Hum. Mol. Genet. 20, 40–50 10.1093/hmg/ddq430 20940149 PMC3000675

[B87] Tofaris G. K. Razzaq A. Ghetti B. Lilley K. S. Spillantini M. G. (2003). Ubiquitination of alpha-synuclein in Lewy bodies is a pathological event not associated with impairment of proteasome function. J. Biol. Chem. 278, 44405–44411 10.1074/jbc.m308041200 12923179

[B88] Vila M. Bové J. Dehay B. Rodriguez-Muela N. Boya P. (2011). Lysosomal membrane permeabilization in Parkinson disease. Autophagy 7, 98–100 10.4161/auto.7.1.13933 21045565

[B89] Webb J. L. Ravikumar B. Atkins J. Skepper J. N. Rubinsztein D. C. (2003). Alpha-Synuclein is degraded by both autophagy and the proteasome. J. Biol. Chem. 278, 25009–25013 10.1074/jbc.m300227200 12719433

[B90] Winner B. Jappelli R. Maji S. K. Desplats P. A. Boyer L. Aigner S. (2011). In vivo demonstration that alpha-synuclein oligomers are toxic. Proc. Natl. Acad. Sci. U S A 108, 4194–4199 10.1073/pnas.1100976108 21325059 PMC3053976

[B91] Wong E. Cuervo A. M. (2010). Autophagy gone awry in neurodegenerative diseases. Nat. Neurosci. 13, 805–811 10.1038/nn.2575 20581817 PMC4038747

[B92] Xilouri M. Brekk O. R. Landeck N. Pitychoutis P. M. Papasilekas T. Papadopoulou-Daifoti Z. (2013). Boosting chaperone-mediated autophagy in vivo mitigates alpha-synuclein-induced neurodegeneration. Brain 136, 2130–2146 10.1093/brain/awt131 23757764

[B93] Yamamoto A. Simonsen A. (2011). The elimination of accumulated and aggregated proteins: a role for aggrephagy in neurodegeneration. Neurobiol. Dis. 43, 17–28 10.1016/j.nbd.2010.08.015 20732422 PMC2998573

[B94] Yamin G. Uversky V. N. Fink A. L. (2003). Nitration inhibits fibrillation of human alpha-synuclein in vitro by formation of soluble oligomers. FEBS Lett. 542, 147–152 10.1016/s0014-5793(03)00367-3 12729915

[B95] Yang Q. She H. Gearing M. Colla E. Lee M. Shacka J. J. (2009). Regulation of neuronal survival factor MEF2D by chaperone-mediated autophagy. Science 323, 124–127 10.1126/science.1166088 19119233 PMC2666000

[B96] Zarranz J. J. Alegre J. Gómez-Esteban J. C. Lezcano E. Ros R. Ampuero I. (2004). The new mutation, E46K, of alpha-synuclein causes Parkinson and Lewy body dementia. Ann. Neurol. 55, 164–173 10.1002/ana.10795 14755719

